# Persistence and resurgence of wild polio virus in sewage samples in Pakistan, challenges, and the way forward: A letter to the Editor

**DOI:** 10.1016/j.nmni.2023.101204

**Published:** 2023-11-26

**Authors:** Mahnoor Fayaz, Ali Aamir Khan, Khabab Abbasher Hussien Mohamed Ahmed

**Affiliations:** Department of Internal Medicine Ayub Medical College, Abbottabad, Pakistan; Faculty of Medicine, University of Khartoum, Khartoum, Sudan

**Keywords:** Polio, Pakistan, Polio vaccination, Mitigation, Public health

Dear Editor,

The poliovirus, a member of the Enterovirus genus and the Picornaviridae family, causes poliomyelitis, or polio. The name poliomyelitis comes from the Greek words ‘‘polio’’ meaning ‘‘grey’’ and ‘‘myelon’’ meaning ‘‘marrow’. This highly debilitating disease kept on inflicting masses until 1988 when polio was eradicated from nearly all countries except for Nigeria, Afghanistan, and Pakistan. Unfortunately, Pakistan has experienced a concerning increase in poliovirus cases over the past two years. While the whole world continues to celebrate polio elimination, countries like Pakistan and Afghanistan continue to live with this never-ending dilemma of polio persistence. Despite continued high standard polio surveillance, certain loopholes in the process have put a hindrance in the eradication of polio in the country. As per the reports of the Pakistan polio laboratory in the National Institute of Health, the first detection of Wild poliovirus 1 (WPV1) has been confirmed in sewage samples from Karachi [1] and two Afghan strains in environmental samples from Peshawar [2]. These findings are highly alarming since only one case of WPV1 was detected in Pakistan in the year 2021. A huge surge in detected cases was observed in 2022 with 20 cases, accounting for a nineteen-fold increase in WPV1 transmission. However, no case was reported from January to March in the year 2022 [3]. In 2023, 3 cases have reportedly been detected indicating a looming fear of a possible resurgence in the endemic nation.

Currently, Pakistan is grappling with the presence of wild poliovirus type I (WPV1), circulating vaccine-derived poliovirus type 1 (cVDPV1), or cVDPV3. The existence of the virus in any area poses significant threats to the health of children and places substantial burdens on the healthcare system and economy of the country. Pakistan faces significant challenges in eradicating the polio virus, with several social factors exacerbating the situation [4]. Detection of WPV1 in an environmental sample from Karachi has given rise to a new polio hotspot in Pakistan besides the already inflicted districts of Khyber Pakhtunkhwa.

In the article by Usman et al. in the Journal of Infections, the authors have rightfully discussed the various reasons that cause a hindrance in the country's polio vaccination drives and the continued official status of a polio-endemic nation by the WHO. Multiple misconceptions regarding the quality of vaccines, their perceived contents, and various religious conspiracy theories have been major reasons behind vaccine refusal and ultimately persistence of polio in this region [5]. According to Wahid B et al., the killing of polio workers in Pakistan is also one of the reasons. One of the contributing factors to this problem is the lack of awareness and misconceptions regarding the dangers of polio, particularly in the northern areas of Pakistan, especially in cities near the Pakistan-Afghan border. In these regions, false religious beliefs are prevalent, further complicating efforts to eradicate polio. Tragically, more than 200 dedicated polio team workers have lost their lives while tirelessly working on polio campaigns. This alarming loss of life highlights the urgent need for increased security measures and enhanced efforts to combat false beliefs and raise awareness about the importance of polio vaccination [4]. Polio eradication campaigns in the region have been unsuccessful also because of uneven geographical distributions of populations with Karachi and Lahore being densely populated have posed significant risks of Oro-fecal transmission and eventual vast spread of the virus.

We highly suggest more investment in the extended polio immunization (EPI) program by the government and the stakeholders. Our Public health infrastructure should be revised and access to EPI centers in the rural areas should be ensured. To combat the lack of awareness among the masses, we suggest the conduction of various awareness campaigns and symposiums that educate the people, especially the ones belonging to the rural areas about the significance of polio vaccination not just in their individual lives but in the country's overall economy too. Constructive use of print and public media can be an extremely useful tool in getting this message across to the vulnerable and hard-to-reach areas of the nation. Prompt detection of polio cases, identification of that specific area and strict surveillance can help us do targeted mitigation of this menace across the region.

## Ethical approval and patients’ consent

As the information used in this study does not include any identifying information from the patients, written consent from the patients was not required.

## Declaration of competing interest

The authors declare that they have no known competing financial interests or personal relationships that could have appeared to influence the work reported in this paper.
